# Explainable prediction of node labels in multilayer networks: a case study of turnover prediction in organizations

**DOI:** 10.1038/s41598-024-59690-4

**Published:** 2024-04-19

**Authors:** László Gadár, János Abonyi

**Affiliations:** HUN-REN-PE Complex Systems Monitoring Research Group, University of Pannonia, Veszprém, Hungary

**Keywords:** Applied mathematics, Computational science, Statistics

## Abstract

In real-world classification problems, it is important to build accurate prediction models and provide information that can improve decision-making. Decision-support tools are often based on network models, and this article uses information encoded by social networks to solve the problem of employer turnover. However, understanding the factors behind black-box prediction models can be challenging. Our question was about the predictability of employee turnover, given information from the multilayer network that describes collaborations and perceptions that assess the performance of organizations that indicate the success of cooperation. Our goal was to develop an accurate prediction procedure, preserve the interpretability of the classification, and capture the wide variety of specific reasons that explain positive cases. After a feature engineering, we identified variables with the best predictive power using decision trees and ranked them based on their added value considering their frequent co-occurrence. We applied the Random Forest using the SMOTE balancing technique for prediction. We calculated the SHAP values to identify the variables that contribute the most to individual predictions. As a last step, we clustered the sample based on SHAP values to fine-tune the explanations for quitting due to different background factors.

## Introduction

Learning from the past gives us the opportunity to correct for mistakes. Machine learning models excel at deriving predictions from real-world data. However, an important element of the learning process is the whitening of prediction black-box models because successful prediction factors need to be known for feedback. The demand for Explainable Artificial Intelligence (XAI) is illustrated by the exponentially growing trend of Google searches^1^. To be completely fair, we have to go further from the explainability to responsible AI’ that is accountable, ethical, transparent^2^ and objective because the successfull predictors may provide information for economic and social decisions.

Using recent advances in network science and machine learning, our objective is to comprehend the factors contributing to staff turnover, specifically focusing on faults in relationships and operational organizational characteristics. To address this, we have developed an explainable machine learning process where the turnover is the outcome variable which is treated as node label in multilayer networks. The purpose is to support future decisions by leveraging insights from the past. Decision support tools often rely on network models, and this article uses information from multilayer social networks of 12 organisations to solve the classification problem and identify possible generalisable factors.

There are numerous distinct reasons why someone might leave an organization. The database under analysis is noisy in terms of specificity, and very small differences are between those who quit and those who stay in SMEs. The complex dynamic system^3^ represented by organizations can appear chaotic and unpredictable. This requires the development of a very high-resolution, sensitive method, which was the aim of our work.

In networks, relationships and node properties can be forecasted. Predicting the likelihood of a future relationship is widely studied and applied in social networks, recommender systems, and anomaly detection^4^. The evolving models in the networks reflect the network evolution by time, has led researchers to develop a number of link prediction techniques^5^. Among others, forecast or recommend a friendship in a social network considering the structure and multiple mechanisms of complex real-world network systems that change over time^6^. These prediction algorithms increasingly consider the hierarchical^7^ and modular^8^ structure of networks in addition to preferential attachment^9^.

In addition to the prediction of links, a common research area in network science is the prediction of labels for nodes. Supervised classification of node properties or classes has been extensively studied because it has an advantage in grouped decision-making on nodes in Web documents where the label indicates the topic^10^, friendship networks, communication networks where the node label can be “fraudulent”, and biological networks^11,12^. These applications typically involve the analysis of single-layer networks.

Node label prediction can be particularly challenging in complex multilayer networked data due to the variety of variables available from raw and engineered data. With an increasing number of layers, various possibilities for prediction arise, including prediction within a single layer or prediction by merging layers. It can be assumed that node properties are link dependent (LD), influenced by connectivity and varying with information propagation through links^13^, or independent of links (LI) altogether. Moreover, in multilayer networks, connections in one layer affect connections in another layer^14^, and supervised learning algorithms can comprehensively consider the information provided by each layer^15^.

In this research, we also investigate which network or operational characteristics have the best predictive power. That is, they show a difference between quitters compared to stayers. We consider 12 small and medium-sized enterprises (SMEs) as multilayer networks^14^ and measured people who quit after 1 year. The termination of employees was interpreted as node label as outcome variable. The article does not consider termination as a node label influenced by a spreading mechanism. The literature discusses the network pattern related to turnovers and usually finds that those with low centrality are more likely to quit^16–18^, which was further investigated^19^.

Hypotheses are frequently used as a starting point when investigating the background factors of the outcome variable. In our data-driven approach, we did not have presumptions, but we sought the strongest predictors and used an explainable machine learning approach. It identifies key predictors from a dataset of relationship deviations from a multilayer network and evaluations that reflect operational or leadership disorders. Using a predictive model, it presents personalized information of influential conditions which lead to the employee’s decision to quit the organization.

The machine learning methodology contributes novel insights into the background of turnover and advances organizational science. In this research fine-tuned background factors of employee turnover was uncovered with the development of the state-of-the-art data-driven approach. The contribution stems from the elements outlined in the following prediction aspects that have not been previously applied together to the prediction of turnover.

1. We analyzed databases from multiple SMEs across various industries, focusing on turnover in a general context rather than in relation to a specific firm.
2. We suppose that there is a deficiency in the literature regarding the analysis of datasets with more than a thousand variables. A multilayer network approach significantly increases the amount of information considered in the feature engineering step. The multilayer network approach to organisations provides an opportunity to further refine and characterize network actors such as the quality of relationships of seekers or brokers.
3. A self-developed data-driven approach was used to select the most influential factors, taking into account the combined predictive power of the variables.
4. The literature rarely discusses the prediction of turnover as unbalanced classes to the best of our knowledge. The number of positive cases is much smaller than negative, although we support machine learning with the synthetic minority oversampling technique (SMOTE)^20^, a balancing technique that significantly increases the accuracy of the prediction^21^.
5. Throughout the process, we strive to maintain explainability because, in addition to the accuracy of the prediction, it is also important for managers and decision-makers to have explanations.
6. We combine the prediction of employee turnover with an explainable machine learning method and use the Shapley Additive exPlanation (SHAP)^22^ technique to measure the marginal contribution of variables to each individual prediction. Common anomaly patterns are uncovered in clusters based on SHAP values to handle the complexity of finding individuals with similarly important predictors. This technique enhances the differentiation of individual situations leading to terminations while identifying common patterns.
7. Finally, we examine the extent to which the factors identified are firm-specific and whether they can be generalised.

This research demonstrates a method for predicting the labels of actors as vertices in organizational networks. Node labels can be not only left out of the organization, but also other outcomes, whose background factors may be of interest to decision-makers.

## Method

To use a popular but similar example, the success rate increases proportionally to the density of the passing network within a football team^23^. The more hierarchical the network, the more deterioration occurs due to the high centralization. To mislead the opponent, it is necessary to facilitate organized communication between team members while maintaining the appearance of disorganization^24^. Among other things, the communication and passing network layers of the network contribute to the success of the team. The evaluation of patterns in the multilayer network of a team may be relevant to estimate the success of the cooperation.

Our goal was to develop a series of machine learning steps that determine the most important predictors of the outcome variable from information obtained from multilayer network connection conditions and organizational, operational characteristics. A model is a simplification of the reality; it retains the essential elements that influence the occurrence of an event. The outcome is the result of a combined effect of several factors in complex systems with many variables. We experienced that the data are noisy and that there are very few differences between classes, those who stay and those who leave the organization. There are questions about whether sufficient information is available to predict classes.

**Figure 1:**
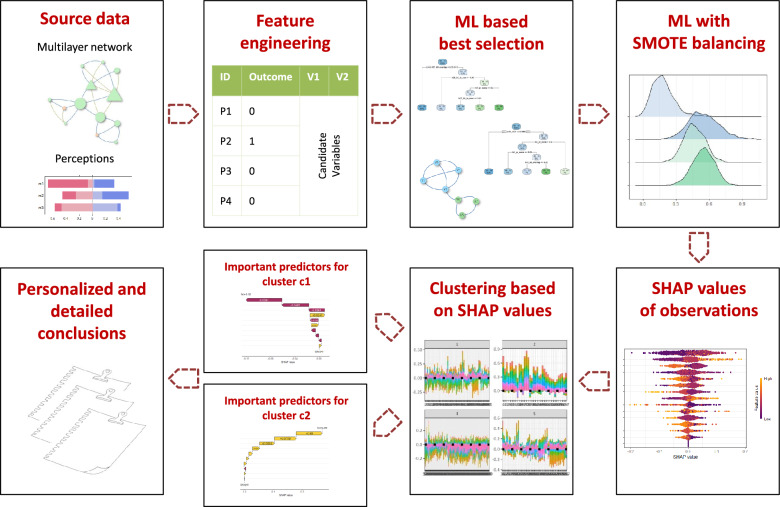
Graphical abstract of this study.

In the first step of the proposed method, we expanded the number of variables in feature engineering, ranked them based on their predictive power, and selected the most influential factors. Prediction was made and for each observation, we individually determined the most important predictors by SHAP value. Based on the similarity of the important predictors, we clustered the employees to obtain the most important factors leading to exit. A visualization is also presented to evaluate clusters. Figure 1 represents the main steps of the proposed data-driven approach. In addition, the pseudo code is given in Code 1. Our method is well-suited for environments where explainability is as important as prediction accuracy to decision-makers.

**Algorithm 1:**
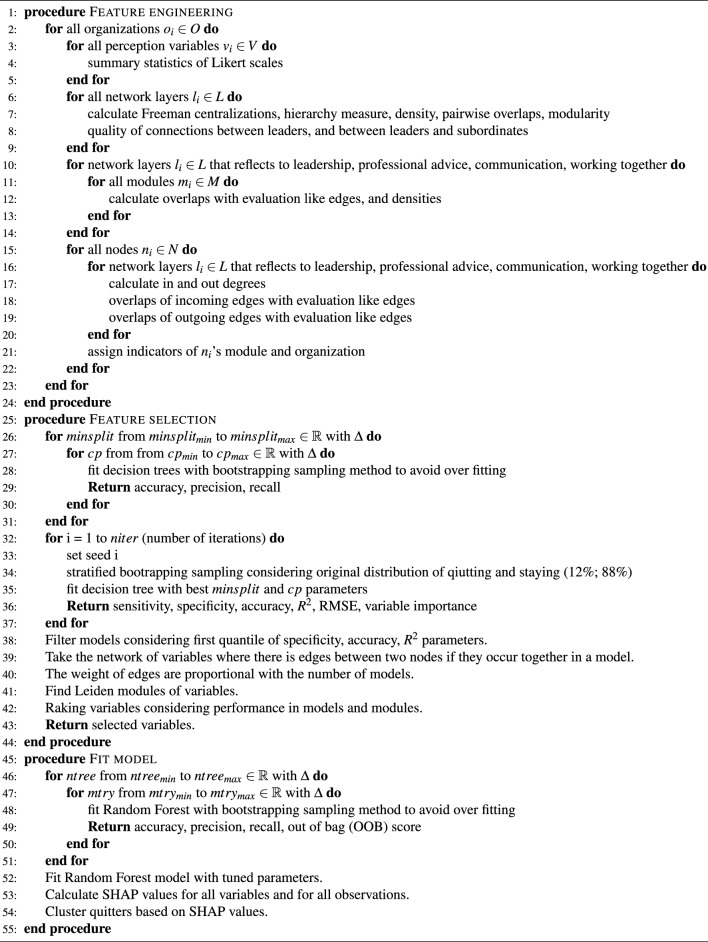
Pseudo code of predicting turnover based on multilayer networks

### Feature engineering

Prediction of node labels requires the development of appropriate individual, mesostructural, and network indicators. Two data sources are available from which additional variables can be developed. Data collection was carried out using questionnaire surveys that were carried out in 12 companies. We used an anonymous questionnaire to measure (1) perceptions of the company (e.g satisfaction, motivational factors), (2) the performance of management and leadership in the organization, and (3) other factors of organization (e.g the information availability, the cooperation of colleagues). Networks were also surveyed by questionnaire. After giving their own name, the respondents were asked the name of a colleague or leader from whom they usually get information, consider a friend, etc. Participation in the surveys was voluntary and participants were informed of the purpose of the research. All researchers worked according to the protocols declared in Code of Ethics of the University of Pannonia, Veszprém, Hungary considering GDPR and Declaration of Helsinki, detailed in the Ethical statement section.

A multilayer connectivity scheme for the nodes in every organizational network is known, from which we can construct layer-wise indicators and layer-overlapping indicators at all three levels of the network structure (individual, mezo structure/moduls, all network). Directed connections define multiple edges between nodes in a multilayer network, in this work multiplex network, and form a multidimensional network $\mathscr {G} = (V, E, D)$, where *V* represents the node set, *D* the set of edge labels defines the dimensions of edges, and *E* denotes the edge set, $E = \{(u, v, d); u, v \in V, d \in D\}$. A multilayer network is a pair $\mathscr {M} = (\mathscr {G},\mathscr {C})$, where $\mathscr {G}$ = $G_\alpha ; \alpha \in 1,\ldots , M\}\}$ is a family of graphs $G_\alpha = (X_\alpha , E_\alpha )$ (called layers of $\mathscr {M}$) and $\mathscr {C} = E_ {\alpha \beta } \subseteq X_\alpha \times X_\beta ; \alpha , \beta \in \{1, \ldots , M\}, \alpha \ne \beta \}$ is the edge set between nodes of different layers $G_\alpha$ and $G_\beta$ with $\alpha \ne \beta$^25^. $E_\alpha$ are called intralayers and $E_{\alpha \beta } (\alpha \ne \beta )$ are referred to as interlayer-connections. The studied intra-organizational networks can be considered to be directed multiplex networks, a special type of multilayer network. Multiplex networks consist of a fixed set of nodes connected by different types of links. In our case, the $G = (V, E, D)$ multidimensional network is associated with a multiplex network with layers $G_1,\ldots , G_{|D|}\}$ where $\alpha \in D,, G_\alpha = (X_\alpha , E_\alpha ),, X_\alpha = V,, E_\alpha = (u, v) \in V \times V; (u, v, d) \in E \; \text {and} \; d = \alpha$}. *D* = 29, *M* = 12, and V ranged between 19 and 132 in this work.

The other source of information are the factors that have been anonymously evaluated in the quality of cooperation, representing the different dimensions of the operation of the network. An assessment $\mathscr {A} = (\mathscr {Y}^{\mathscr {O}})$ is a set of evaluations along all dimensions where $\mathscr {Y}^{\mathscr {O}} = \{y_t; t \in \{1,\ldots , T\}\}$, *T* is the number of topics evaluated in questionnaire, and $y_t = y^i_t; t \in \{1, \ldots , T\}, i \in \{1, \ldots , N^{o_j}\}$ vector represent the answers of members of *M*==$o_j$ i.e. $j{\text{th}}$ organization, where the maximum number of respondents is $N^{o_j}$ is the number of member of $j^{th}$ organization. Due to the nature of the survey, *i* is anonymously evaluated and cannot be related to the links in the network.

The information content of the two data sets can be linked because they are about the same network. The output of multiple models is better than using a single model^26^. Both data sources were used to generate the set of variables.

The anonim evaluations was treated as information for the network as a whole. Negative and positive responses was separated for all topics. From the six-point Likert scale scores, we calculated the proportion of responses 1, 1 and 2 and 1,2,3 relative to the total number of responses, which were then created as variables. We did the same for positive responses. The proportion of ’not relevant’ responses was also treated as a variable, as it may have information content in the prediction.

To characterize the extent to which network layers are centralized, we used Freeman centralization (degree, betweenness, closeness)^27^ to uncover how centrality affects group processes. A hierarchy measure based on the local clustering coefficient^28^ was applied for each network layer to calculate the hierarchy, which was successfully applied in other research^29^. Hierarchies are more difficult to form in sparse networks^30^ and density related to the spread dynamics of information at network^31^ and local level^32^. Among the network-level indicators, we counted the density of each network layer. Local and global overlapping edge dimensions related to appreciation and trust. Sympathy factors alongside communication relations indicate deeper relational dimensions^14^.

The variables were generated from deeper parts of the network structure. The performance and satisfaction of the network nodes also depend on smaller communities at the mesostructure level. Small communities were defined on the basis of relationships in the network and not on the basis of the artificial partitioning of networks (organizational units). We identified Leiden modules^33^ with a higher density of connections than in the rest of the network in a network layer representing cooperative relations. We calculated the density of connections in communities in every network layer. Consistency indicators of modules in all network layers were generated considering the proportion of directed edges originating within a module and ending within the same module. Lastly, at the mezostructure level, meaningful overlap measures were derived considering relationships within modules. All mesostructure-level indicators have been joined to individuals, indicating the quality of cooperation in the close community. We assigned the same indicators to the nodes that belong to a community.

Network science provides a number of metrics for generating node characteristics, from which several variables are calculated. Degree and centrality are widely used individual indicators. In each network layer, we counted nodes’ incoming and outgoing degrees. The absolute value of the PageRank centrality^34^ and the normalized Min–Max rankings of all network layers are in the database as candidate predictors. PageRank centrality is a good indicator of the importance of actors in the network. Reciprocal relationships indicate deeper cooperation, trust, and agreement. Reciprocity rates were calculated for each network member of every network layer. The reciprocity ratio shows the proportion of mutual candidates.

### Feature selection

Using 1500 variables, we look for factors that are good predictors of belonging to positive (1) for leavers and neutral (0) for stayers classes. The aim of feature selection is to improve the prediction performance using data extraction, including preparing understandable data^35^. The finding of fast and cost-effective predictors is also an objective of selection considering an objective function^36^. In addition to the two general objective functions of maximum goodness of fit and minimum number of variables, a third is formulated. We have chosen the Random Forest procedure; therefore, we consider decision trees for which a small set of variables cooperated perform well in the classification process.

We wanted to select the most useful variables based on an information criterion to build the classification model. With all its advantages and disadvantages, we use a hybrid procedure^37^ among the unsupervised feature selection procedures. Spectral dimensionality reduction procedures (such as PCA, MDS) are not an option in this work because they may produce predictors that are difficult to explain. Correlated variables provide little information for prediction and may pose a problem^38^ therefore similarity-based dimensionality reduction^39^ methods are not appropriate in this article.

The following procedure is used to find variables whose variances differ between the classes to be predicted.

1. The data set has an effect on the selection of features^40^. In this work, taking advantage of the impact of sampling on the instability of feature selection, we generated 25,000 train data set using the random sampling with replacement method (bootstrap)^41^ with preserving the stratification of the outcome variable. 12% of the training set is quitter, same as the sample. All 25k train set consist of 399 rows, which is the 70% of the data set. Then we perform 25,000 classification with decision trees. The decision tree uses an information gain function^42^ to select the variables that give the most accurate classification at each decision step of the tree when partitioning with a complexity control^43^.
2. We selected classification trees with a sensitivity (true positive rates) of at least 55.9%, accuracy of at least 74.5%, $\text{ R}^2$ of at least 29.2% on the train set. These measures were the thresholds of the first quantiles (Q1). 10. 1% of the classification trees reached the Q1 threshold for each criterion. Specificity (good prediction of 0 class) has not been shown to be a good measure of selection, as the outcome is skewed; therefore, most decision trees have a specificity above 90%.
3. The variables of the best-performing decision trees were networked, where the nodes are the variables, and there are edges between them if they were in a common well-performing decision tree because they act cooperatively. In the predictor network, we uncover Leiden modules^33^ to identify groups of variables that frequently co-occurred.
4. We selected the predictors with the best performance based on how often they were in the top 5 in terms of importance considering the cooccurrence-based module categories from Step 3. We selected 28 variables with this step, but we wanted to make further reduce the number of variables.
5. The best selection of the variables was determined by fitting with the selected variables using a sequential replacement procedure. We selected the model with the number of variables with the best adjusted $adjR^2$ and Mallows’ cp, which is widely used in model selection. The SMOTE balancing procedure^20^ for sampling was used due to the skewness of the sample and we found that it increases the sensitivity of the fit while maintaining accuracy. This step was performed in R using a regsubsets function in the leap package.

These steps provided the selection of the 12 variables that jointly perform well in the classification.

### Machine learning with SMOTE-based balanced data

Several machine-learning methods are available to solve classification problems. The two most commonly used are boost (Adaboost^44^, XGboost^45^) and bagging (Random Forests^46^). For some problems, the performance of the two methods is very similar^47,48^. Combining a large number of trees improves prediction accuracy^41^, therefore, ensemble methods are popular.

We do not aim to compare the methods in detail because this is beyond the scope of this work. Boosting methods perform well to solve binary classification problems, but their parametrization gives many options and this affects their performance. The Random Forests procedure also has several advantages^49^. Robust to small perturbations in the data. The column subsampling method used in Random Forests has the advantage of avoiding overfitting. In addition, a Random Forest is easier to train and tune. A bootstraping method was used as cross validation to optimize the model complexity. In addition to the Random Forest method, we tested the classification using Adaboost, Extra Trees, and Logistic Regression methods. Methods obtained very similar results, however, Random Forest outperformed the other three using the same train and test sets. Results are summarised in Table 2. Interestingly, almost the same positive cases were misclassified by methods. The identically false-negative classifications raise the problem of predictability or appropriate information for prediction, which would stretch the boundaries of this article.

A fast implementation of Random Forests in ranger package^50^, adabag package for Adaboost, ranger package for extratrees and glm function for logit in R was applied to perform classifications. SMOTE sampling method was used to address the imbalanced class distribution and enhance the specificity. Using SMOTE the model is much more sensitive to minor class. We use the smotefamily package implemented in R for sampling during training.

### Explain model with SHAP values

The crucial parameter for solving real-life problems with machine learning is making the model explainable if decision makers want to use the information obtained for interventions. An accurate model alone is useless if it remains a black box, and we cannot explain to decision makers the antecedents of leavers’ past decisions. There are several research that aims to provide explanations and it is one of the most important research areas in recent years because explainability increases confidence in the results.

Establishing explainability has started with several ideas. One of them identifies the most influential factors by analyzing differences in important variables extracted from various classification procedures^51^. Explainability can be achieved by extracting information from decision rules and analyzing them statistically^52,53^, but it is an AdaBoost-specific approach, however, outperforms model-agnostic explanation methods.

Two widely used model-agnostic frameworks have been recognized as XAI tools to let domain experts see the reason why a black-box model gives a certain output. The number of citations in Local Interpretable Model-Agnostic Explanation (LIME)^54^ and SHapley Additive Explanations (SHAP)^22^ indicates their similar prevalence. Evaluation of their performance in the context of credit risk management shows that SHAP has a greater ability to define different groups of observations^55^.

The SHAP is a special adaptation of a concept coming from game theory. It measures the contribution of each explanatory variable to each prediction and can be assessed regardless of the underlying model. SHAP values that express model predictions as linear combinations of binary variables that describe whether each covariate is present in the model or not. SHAP method approximates each prediction *f*(*x*) with a $g(z')$ with a linear function of binary variables $z' \in \{0,1\}^M$ and quantifies $\phi _i \in \mathbb {R}$ defined as Eq. (1).

1 $$\begin{aligned} g(z') = \phi _0 + \sum _{i=1}^{M}\phi _i z'_i \end{aligned}$$

where is is the explanation model, $z'$ is the coalition vector with 0 or 1’s, M is the number of explanatory variables the maximum coalition size and $\phi _i \in \mathbb {R}$ is the attribute of features for the feature *j* i.e. Shapley value. For example if an $x'$ coalition vector is the instance of interest is a vector of 1’s (all feature are present, the Eq. (1) simplifies to Eq. (2)

2 $$\begin{aligned} g(x') = \phi _0 + \sum _{i=1}^{M}\phi _i \end{aligned}$$

The Shapley value is the average of all marginal contributions to all possible coalitions^56^. The Shapley value is an estimation with Monte Carlo sampling^57^, because the evaluation with and without features *j* exponentially increases the number of possible coalitions. The Shapley value expresses for each prediction the deviation from their mean by the predictors, that is, the contribution of the variable *k* in the prediction of instance *l*. The SHAP importance of a variable *k* representing the added value or the impact of the prediction relative to the other characteristics is the average of the SHAP values of the variable *k* as shown in Eq. (3).

3 $$\begin{aligned} I_k = \frac{1}{n} \sum _{i=1}^{n} |\phi _j^i| \end{aligned}$$

where *n* is the number of observations, *j* is the feature of interest.

Two data tables are available for further operations and visualizations as a result of the calculation of SHAP values. One contains the characteristic values for each observation. The other is the SHAP values for each variable and observations. These two tables can tell us if a feature is more likely to increase the risk of turnover and the extent of the influence on the outcome of the classification.

### Improve explainability by clustering instances based on SHAP values

The complexity of the data can be observed in that by selecting the most characteristic factors and establishing explanatory power, contrasts can still be detected in the high variance of SHAP values that show a large variance even for positive cases. It suggests that a positive outcome, the turnover, can occur with a wide range of antecedents. This realization inspired us to find common patterns in the cases.

We aim to resolve the complexity by unsupervised clustering. Clustering of SHAP values is relatively rarely discussed in the literature. In the health field, it is used to explore the different subgroups at risk of re-attendance^58^, in the insurance field to understand consumers’ needs^59^, in the environmental field insights on the provincial-specific decarbonization strategy^60^, and to detect concept drift using manufacturing data^61^. Each of these works reflects the specificity and aims to better understand smaller groups of instances.

The goal is to enhance the global interpretability of the prediction model provided by the post-model SHAP explanations. Local interpretability can be achieved with independent clustering explanations for individuals^62^. Clustering of instances based on SHAP values leads to groups in which similar predictors contributed to the prediction of members’ classes. Clustering only applies the SHAP value without considering original feature values. However, feature values matter in the description of clusters. Clustering can be done without further scaling of SHAP values.

In this work, we focus on the positive class, those who leave the organizations. The Partitioning Around Medoids (PAM) algorithm^63^ was applied to identify clusters because it has the robustness to outliers^64^ and performs better than the popular k-means algorithm^65^. Euclidean distance and the Silhouette method were employed for cluster evaluation. The Silhouette width assesses the cohesion of members in clusters, serving as a widely used validation metric that aggregates the similarity of an observations to their own cluster relative to their nearest neighboring cluster. With a range from − 1 to 1, higher values signify better clustering. The average Silhouette width was calculated for varying cluster numbers, ranging from 2 to 10, using the PAM algorithm. Six clusters demonstrated the highest Silhouette width value.

### Similar works

The study of turnover is a frequently researched topic given the challenges and expenses associated with replacing workers. In the literature on employee turnover, researchers have long analyzed the impact of both external (labor market conditions) and internal (job satisfaction) factors on an individual’s decision to leave their job^66^, often through the lens of signature models.

The unfolding model^67^ delves deeper into the decision-making process behind voluntary employee turnover, identifying triggering factors. By incorporating both market-pull and psychological-push elements, this model provides a better understanding of why employees choose to depart organizations. Focused primarily on psychological processes and external events, the unfolding model sheds light on the intricate dynamics that influence employee decisions.

The theory of proximal withdrawal states^68^ bridging content models and process models like the unfolding model into a single model. Unlike the unfolding model, it emphasizes the role of sudden shock-like factors, which often prompt departures sooner compared to those driven by dissatisfaction^69^. By delineating the withdrawal states of employees, the model identifies various motivational factors contributing to termination. Job embeddedness^70^, rooted in social networks^71^, also plays a significant role in the theory of proximal withdrawal states, describing the driving forces behind why individuals decide to stay in the organization. The comprehensive approach of the model holds great potential to inspire further research on termination analysis, with some clarification points to consider^72^. Furthermore, as computational capabilities, along with machine learning and network science methods, continue to evolve, there is still an opportunity to overcome statistical limitations and extend the applicability of these models^73^. Various predictive algorithms have been developed to forecast turnover and improve business performance. In the following sections, we review research conducted by different authors, organized according to the machine learning steps described in the Methods section.

*Step 1 Variables worth considering in the research* The attrition of highly skilled and embedded workers in the healthcare sector has been explored through the analysis of data from 12,000 workers using various machine learning algorithms^74^. Overtime was identified as the most significant variable in this context^75^. The likelihood of turnover decreases with increases in salary or decreased overtime; however, work-life balance, job level, years with the company, and years with the current manager are identified as the four main factors influencing turnover^76^. ML models ranking factors revealed a positive correlation between turnover and home-workplace distance, a negative correlation with age, and a proportional correlation with salary^77^. Another study highlighted that individuals who do not receive promotion, long work hours after a promotion, or no pay increase tend to leave^78^. Using various classification algorithms, it was concluded that avoiding monotony and introducing workplace challenges are crucial factors in reducing turnover^79^.

The network science toolbox provides valuable tools for predicting terminations and uncovering underlying explanations. Structural equivalence^80^, as well as importance/centrality in communication^16^ and friendship^17^ networks, have proven to be effective predictors in certain companies. When considering two companies and two types of networks, a logit model has been employed to enhance the precision of turnover prediction^18^. Centrality, particularly broker and structural hole roles, is associated with social capital, and higher centrality values reduce the likelihood of turnover. This association has been observed in project management and research development business departments^81^. An in-depth social network analysis, accounting for the overlap between advice and workflow relationships, has provided additional explanatory factors^19^. High in-degree in the advice network (indicating high centrality) is identified as a high risk for turnover, in contrast to peripheral actors. A three-way correlation between centrality, social support, and turnover has been demonstrated by the Pearson correlation^82^. Various studies in this field report correlation coefficients (r values) between turnover and centrailty ranging from 0.11 to 0.65 across different organizations. Furthermore, internal networks are more commonly studied to predict turnover, the importance of external networking should not be overlooked^83^.

*Step 2 Feature selection.* The identification of explanatory factors of turnover is crucial for companies aiming to improve managerial decision-making. In pursuit of this objective, various techniques were applied to the Kaggle IBM dataset^84^, including the Recursive Feature Elimination algorithm, Mutual Information, and Meta-heuristic algorithms such as Gray Wolf Optimizer and Genetic Algorithm. Additionally, the Best-Worst Method was employed^76^. Then the ML was applied to test the predictive power of selected variable. In another study, the Intensive Optimized Principal Component Analysis (PCA) was utilized for feature selection before applying Random Forest (RF) to unveil turnover-associated factors. The data source for this study was an enterprise resource planning (ERP) software database^85^, and the number of variables was fewer than 100.

*Step 3 ML methods.* In the analysis of the Kaggle employee attrition dataset^84^, a combination of machine learning methods (Support Vector Machine, Gaussian Naïve Bayes, K-Nearest Neighbors, and Neural Networks) and survival analysis was employed to identify the top contributing attributes, focusing on the primarily demographic and work-related features among the total of 34. This analysis, especially crucial for at-risk departments^75^. They determined that the Logit model yielded the best performance. Another study on the same dataset found that Random Forest (RF) was the most effective model^86^. In the context of a big data company, RF outperformed other algorithms, including Logistic Regression, K-Nearest Neighbors (KNN), and Decision Tree, in modeling turnover^87^. A comparison involving Decision Tree, RF, k-nearest neighbor, and Naïve Bayes classifiers on a dataset consisting of 15,000 observations also identified RF as the best-performing model^78^. Ensembles algorithms, such as XGBoosting, gradient boosting, and RF, were found to generally outperform other classifiers across various datasets^88^. Additionally, XGBoost was identified as the top-performing method in an analysis of data from an HR Information Systems, which was characterized by noise and susceptibility to overfitting. The boosting technique employed by XGBoost enhances training for challenging-to-classify data points and incorporates excellent inherent regularization^89^.

*Step 4 ML-based handling of unbalanced prediction problems.* In the prediction of nurse turnover, the application of SMOTE significantly improved the accuracy. This prediction was based on eighteen carefully selected demographic and work-related categorical variables, utilizing the logit, Random Forest (RF), Decision Tree (DT), and XGBoost algorithms. Notably, the RF model exhibited superior performance^90^.

*Step 5 Enhance explainability.* The authors propose a transparent AI implementation framework utilizing LIME in the prediction of turnover. This framework serves as a valuable resource for HR managers, aiming to enhance the interpretability of AI-based machine learning models. The use of LIME aids in addressing trust issues related to data-driven decision-making^91^. This implementation was demonstrated using the Kaggle IBM dataset^84^.

Research on turnover has been extensive, witnessing the emergence of numerous machine learning methods. In the realm of network factors, centrality has predominantly been explored as a precursor. Many studies typically focus on one or two organizations, enabling the identification of firm-specific factors but limiting generalizability due to diverse organizational contexts. Recognizing the need for decision-makers to understand their specific firm’s factors, HR analysts increasingly employ ML tools for turnover analysis. Multilayer network modeling of organizations is notably scarce in the literature, highlighting a gap in understanding turnover based on multilayer network factors.

In our work, we contribute to turnover research by analyzing a comprehensive dataset encompassing 12 organizations. These firms are treated as multilayer networks, described by 28 network layers, providing insight into the network position of individuals who quit. Workplace factors, such as satisfaction levels, are measured through an anonymous questionnaire, offering a wealth of data for turnover analysis and generalization. The analysis pipeline we present for turnover analysis represents a novel contribution.

## Results of the goal-oriented application of method

In this section, some specific information on the data analysis pipeline described in the methodology is presented, including the prediction results provided by the ensemble model. The method is suitable to solve almost any classification problem. In this research, we apply the method to the prediction of node labels. The label in our case can take two values: 1 if the worker leaves, 0 if he stays.

In the following subsections, we examine the variables to be considered from an organizational perspective at the organizational, close co-worker, and individual levels. We will then examine the selected variables and their meanings obtained as a result of the feature selection. The prediction results are then presented. Finally, focusing on the cases of quitting, we analyze in more detail the factors influencing employee turnover.

### Organizations and data collection

We surveyed 12 SMEs and engineered a set of characteristics at the organizational, mesostructure, and actor levels to predict turnover. Two surveys were carried out in 2021–2022 using online questionnaires in all SMEs. One of the surveys anonymously evaluated perceptions of satisfaction, motivation, loyalty, and leadership perceptions on a Likert scale of the operational characteristics of organizations. In the other questionnaire, we asked the respondents to name their relationships with colleagues, professional advisors, and confidants. The respondents also asked for the names of the staff members or leaders who are highly valued and considered key people. The second questionnaire allows us to represent a multilayer network of organizations in which relationships are multidimensional, with evaluative layers in addition to relational layers. One year after the surveys, we asked the HR managers of the SMEs to name those who had resigned of their own intention. Data from the SMEs surveyed are summarized in Table 1.

**Table 1:** Background data on the SMEs surveyed.

Id	Industry	Number of members	Number of respondents	Number of leavers	Number of members in the sample	Number of leavers in the sample
1	Energy	49	44 (90.0%)	2 (4.1%)	43	2
2	Engineering	45	41 (91.1%)	6 (13.3%)	35	6
3	Engineering	132	116 (87.9%)	9 (6.8%)	119	8
4	Health	34	29 (85.3%)	0 (0.0%)	26	0
5	IT	45	43 (95.6%)	4 (8.9%)	40	3
6	IT	19	17 (89.5%)	5 (26.3%)	17	5
7	IT	33	31 (93.9%)	7 (21.2%)	26	6
8	Manufacturing	113	72 (63.7%)	2 (1.8%)	81	2
9	Manufacturing	22	18 (81.8%)	6 (27.3%)	14	3
10	Manufacturing	74	63 (85.1%)	8 (10.8%)	60	8
11	Manufacturing	72	49 (68.1%)	9 (12.5%)	56	8
12	Trade	83	66 (79.5%)	21 (25.3%)	57	19
Overall		721	589 (81.7%)	79 (10.96%)	574	70

Organizational members do not hold leadership positions within the organization.

The lower response rate for SMEs in the manufacturing and trade industries is due to a higher proportion of blue-collar employees. Participation or rejection rates in the survey was included as a feature in machine learning. The average turnover rate in the SMEs surveyed was 10.96%, which is consistent with others^19^. The proportion of quitters increases as the size of the companies decreases on average. Taking into account the employment status, the analysis database comprised 574 respondents who has no leadership position. Of these, 70 individuals voluntarily terminated, which is 12.2%. The observations included individuals who did not respond to the questionnaire, but their information on network structure could be assessed because other respondents could mark them on the questionnaire.

### Variables to be considered for prediction

#### Features at organizational level

The anonymously measured variables on the functioning of the organizations and their management are considered as perceived indicators of the network. For the 79 Likert scale variables, we computed averages, the proportions of response categories 1-6 (both negative and positive), and the proportion of irrelevant responses. This process resulted in 869 candidate features for machine learning, adding 790 extra features to the dataset.

The knowledge creation and spreading of the effectiveness of communication channels are strongly influenced by the degree of hierarchy in the organizational structure, which affects staff morale and performance. Hierarchy is particularly present in military and educational organizations with disadvantages^92^, and knowledge-based organizations must strive to break down hierarchies and develop organic organizations by incorporating higher relational dimensions^93^. Dyadic and group perceptions lead to a variety of forms of social hierarchies and are not necessarily based on dominance relations, although individuals navigate multiple social hierarchies simultaneously in overlapping social networks^30^. The measurement of hierarchy and centrality are the two main perspectives to quantify the influence and distribution of social resources^94,95^.

We calculated the proportion of the edges of the relationship that include the evaluation dimension at the organizational level.

We calculated several ovelapping measure at the organizational level which indicate the proportion of the evaluation like edges occur together relationship type of edges. For example, in addition to communication network connections, the frequency of marking the same persons in the “motivates me” theme.

We calculated several overlapping measures at the organizational level, which indicate the proportion of evaluations where edges occur together, representing different relationship types. For example, in addition to communication network connections, we examined the frequency of individuals being marked in the theme ’motivates me’ together with communication using multilayer nature of organizations^14^.

#### Features at close co-worker level

Operations take place in small communities in organizations. In networks, modules represent denser relationships with a higher density of relationships within modules than outside modules. The candidate features include the degree of overlap between relational connections and evaluations within the module. We counted the consistency of modules by indicating the proportion of directed edges started from a module member that end within a module.

Leader-leader exchanges (LLX) and trust have the largest effect on project and organization performance^96,97^, and explain job satisfaction^98^. Indicators (density, overlap) of the quality of relationships between leaders were included among the characteristics of the candidates. Based on preliminary qualitative research, we distinguished between SMEs where conflict between managers could be identified due to human relations or professional reasons. We found that communication relationships between managers were significantly less likely to overlap with the dimension of good leadership evaluation.

#### Features at actor level

The most basic and widely used network structural metrics are degree and centrality. Leader-member exchange (LMX) is affected by the structural differences of individuals measured by the degree of centrality in the advice network^99^. LMX has a reciprocal correlation with job satisfaction^100^ and turnover^101^. The ranking position in the communication network^16^ and the friendship network^17^ has an important effect on the turnover decision.

The emotional and friendship dimensions of relationships influence the LMX^102^. The so-called “density of multiplex networks” indicates the cohesion of management and affects the team performance^97^. We interpret the density of multiplex networks as the overlap of network layers^14^, which indicates coupled ties between dyads. The aggregated overlap indicators for a group or organization is made up of individual indicators, as described in the previous subsections. Candidate predictors include the degree of overlap in ego networks, reflecting the perceived competencies, skills, and trust relationships of employees with their own manager(s) and vice versa.

Co-operation between individuals can also be viewed as an exchange^103^. Mutual relationships are strong and indicate emotional intensity and intimacy^104^, especially in trust and friendship networks. We include the rate of reciprocity of individuals’ outgoing and incoming relationships as candidate variables to examine the effect of the proportion of strong relationships on turnover.

### Feature selection

We sought the mathematical model that best captures reality and predicts the turnover. Based on the relevant literature and available data, we have created 1528 candidate variables for the prediction model, from which we need to select the strongest predictors that cooperatively provide the best classification. The variables include network-level indicators that arise from perceptions of cooperation and human-relationship dysfunctions. The characteristics of a close group indicate the quality of cooperation between a close team. In addition, individual indicators related to position, embeddedness, skills, competencies, acceptance, evaluation, trust, and friendships. Network-level indicators are categorical in our data because the variable can take as many values as the number of SMEs under consideration, in our case 12. The description of selected variables are in the Supplementary material.

**Figure 2:**
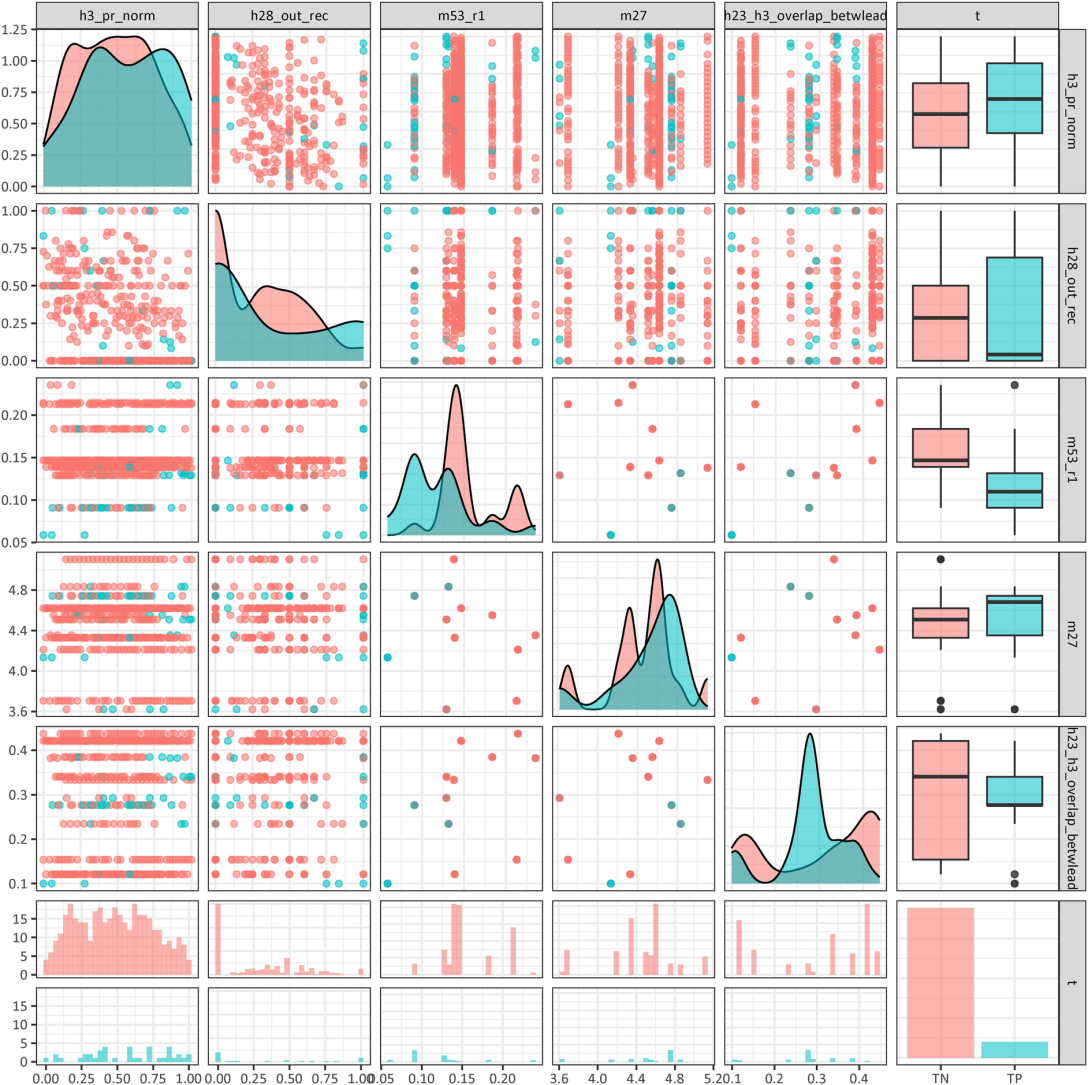
Important predictor variables represented the data and the challenge of separation/classification (True negative (TN): stayer who can predict well; True positive (TP): individuals who quit the organization and predictable).

Organizational-level indicators can also be interpreted as categorical variables, taking only 12 different values. However, they still provide a good characterization of the working environment of the network actor through the ratings of the whole group, whether he works in a company where the cooperation system is adequate or not. In Figure 2, 5 of the selected variables are presented, showing their distribution by class. The distributions are very close to each other, so in anticipating the model-building step, we have narrowed the plotted sample to show those correctly classified by the model. The model finds the differences between classes using the right combination of variables.

The bootstrap sampling for training set and the fitting of 25,000 decision trees took 6 h on an Intel(R) Core(TM) i7-2600 CPU @ 3.40GHz, 3401 Mhz processor, utilizing only one core. Each fitting involved a stratified bootstrap sampling technique that preserved the stratification of the outcome variable, which means that 12% of sample was quitter. Each decision tree was fitted using the rpart function within the rpart package in R, which is an implementation of Classification and Regression Trees^105^. RStudio was applied in Windows 10.

### Prediction of the employee turnover

Given the database for machine learning classification, containing known stayers and leavers, moreover, 12 well-performed variables in addition to the outcome. The number of observations was 574 from which the number of positive cases was 70 (12.2%). For the classes, the database is skewed, so during the training step, we used the SMOTE procedure to rebalance the training sample, aiming to enhance the accuracy of the model in classifying the positive classes. The variables are not correlated with each other, and all are important for prediction, and acting jointly, they can give accurate results in decision trees as detailed in the Method section.

We used a non-linear ensemble model for classification because the distribution of variables between classes shows that they are very similar (Fig. 2). In addition, elements in the distribution of variables by class are difficult to explain. For example, according to the organizational indicator m53_r1, stayers are in an organization with a higher proportion of perceived inequity in the distribution of delegated work. Similarly, for the m27 variable, stayers’ organizations are, on average, rated as worse. It is difficult to explain the drivers of quit organizations.

Tuning the parameters of the Random Forest model is much simpler than the boosting procedures, in which several parameter adjustments are required to obtain the most accurate prediction. Bootstrap (sampling with replacement) cross validation was employed for the optimal complexity of model. The number of trees and the number of random variables are the two influencing parameters that have the greatest impact on the accuracy of the model. For the number of trees (ntree), we considered the minimum number above which the accuracy of the model for the train set. The decay of the error did not occur for more than 200 trees. We observed that the accuracy on the test set also and it did not changed with the increase of ntree. For the number of variables (mtry), we considered the out-of-bag error (OOB), which was calculated for each possible number of variables. The minimum value was obtained at 5, i.e., the model works most accurately when five variables are included. The OOB value was 0.0934.

For the model, 70% of the sample was the train, and 30% was the data test set. The distribution of classes in the train and test databases was the same as the initial distribution; that is, the neutral cases were 88% and positive cases 12%. In the training step, the training database was balanced using SMOTE, which increased the specificity (the prediction accuracy of positive cases). We found in the fitting of several models that the use of SMOTE resulted in a 10% increase in sensitivity. As the work aimed to develop a method for detecting background factors in positive cases, this was a particularly important methodological step.

The Random Forest (RF) performance was benchmarked against other ensemble machine learning (ML) methods. Bootstrap cross-validation was employed to prevent over- or under-fitting, ensuring an optimal model complexity. Fine-tuned parameter models were fitted to the same training database to maintain comparability. RF is a widely-used technique for turnover prediction and machine learning in general. Adaptive Boosting (Adaboost)^106^ is another popular ML method, primarily for binary classification, learning from weak classifiers’ mistakes. It is not prone to overfitting, but it is sensitive to noisy data. The Extremely Randomized Trees method^107^ is similar to RF but differs in that it does not resample observations when building trees (bagging) and “best split” of predictors is used after a randomly chosen split-point. In this study, we tested the performance of this method with enabled as well as disabled bagging.

The results are summarized in Table 2. It is observed that the methods can identify quitters in the test set to a similar extent, albeit with varying proportions of false positives. This discrepancy can be considered a cost of classification and impact the accuracy. RF identified as the least costly method. The fitting of each models on an Intel(R) Core(TM) i7-2600 CPU @ 3.40GHz, 3401 Mhz processor, utilizing only one core using RStudio in Windows 10 was very fast, less then 5 seconds. This is not a time-consuming task for a database of this size. Performance parameters were calculated with confusionMatrix function in caret package^108^ in R. Confidence interval (CI) for precision were calculated by a procedure^109^ which evaluates whether the overall accuracy rate is greater than the rate of the largest class^108^.

**Table 2:** Evaluation the performance of various machine learning methods.

	Random forest	Adaboost	Extra trees enable bagging	Extra trees disable bagging	Logit
Number of TP	16	15	16	16	14
Number of FN	5	6	5	5	7
Number of FP	24	43	36	32	27
Number of TN	127	108	115	119	124
Accuracy	0.8314	0.7151	0.7616	0.7849	0.8023
Precision (95% CI)	0.7667–0.8841	0.6414–0.7812	0.6908–0.8232	0.7159–0.8438	0.7349–0.8590
Specificity	0.8411	0.7152	0.7616	0.7881	0.8212
Sensitivity	0.7619	0.7143	0.7619	0.7619	0.6667
F1	0.5246	0.1899	0.4384	0.4638	0.4516
AUC	0.8250	0.7650	0.7950	0.7870	0.7980

*TP* true positive, *FN* false negative, *FP* false positive, *TN* true negative cases in contingency table.

The Random Forest performed best, and we can find actors in networks who can be labeled with a positive class label. However, this information is not satisfactory, we have information only about actors with positive class, but there is no information about the reason. In the next step, it is necessary to find possible explanations for the classifications. As described in the Methods section, we make the model explainable by calculating SHAP values.

**Figure 3:**
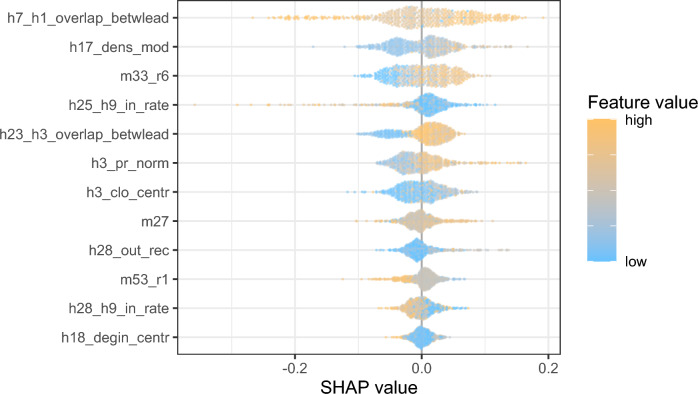
The distribution of SHAP values by variables across the entire dataset. The SHAP values are on the x-axis with a slight jitter to avoid overlapping points. Variables are on the y-axis. The order of variables is proportional to their contribution to the classification. The points represent individuals in the data. The color of point depends on the value of the data point in the variable.

SHAP values are plotted in Figure 3. The beeswarm figure shows four sets of information. (1) The order of the variables on the left is proportional to the relative contribution of the variable during prediction. The higher it is listed, the greater its contribution relative to the other variables with respect to Equation 1. (2) The dots present the SHAP values of the individuals for each variable on the horizontal axis. An individual is plotted as many times as the number of variables. To avoid overlaps, the plotting technique uses jitter. (3) If the SHAP value is negative, the variable predicts a negative class for the individual, and if positive, it predicts a positive class. (4) The color of the dots is proportional to the characteristic value of the individual, as explained in the legend.

Individual indicators slightly explain the model. For the variable h25_h9_in_rate, a lower value predicts quitting, i.e. the less the cooperators perceive the individual as a key person, the more likely he or she is to quit. However, this is not true for all actors with low values. For the h3_pr_norm Pagerank centrality indicator, the less important one is in the communication network, the more likely it is to quit. However, there are already discrepancies in the friendliness indicators. In the case of the variable h28_out_rec, which indicates the proportion of strong friendship relations with respect to the outgoing edges of the friendship network with the rate of reciprocal connection. Both high and low feature values predict quits. The overlap of incoming cooperative relationships with the friendship in individual incoming edges h28_h9_in_rate confusingly predicts the positive class of the individual.

For organizational indicators, even more, contradictions are found; that is, termination occurs in organizations with good and poor indicators. The most important predictor is an organizational-level indicator (h7_h1_overlap_betwlead) related to deep cooperation between leaders. The high feature value predicts both the positive and negative classes, and low feature values are associated only with low SHAP values. The second important factor related to the intensity of coordination activity between close coworkers (within the module) h17_dens_mod predicts exit with higher probability at a high feature value indicating that the overmanagement among close colleagues leads to turnover at a higher probability. Organizational indicators seem not to be a good predictor alone.

The inconsistencies suggest that several phenomena are present simultaneously in the database. This is realistic given the diversity of organizations in the database and the variety of human intentions. The complexity of this variation requires decomposition and finding similar instances.

### SHAP value based clusters

**Figure 4:**
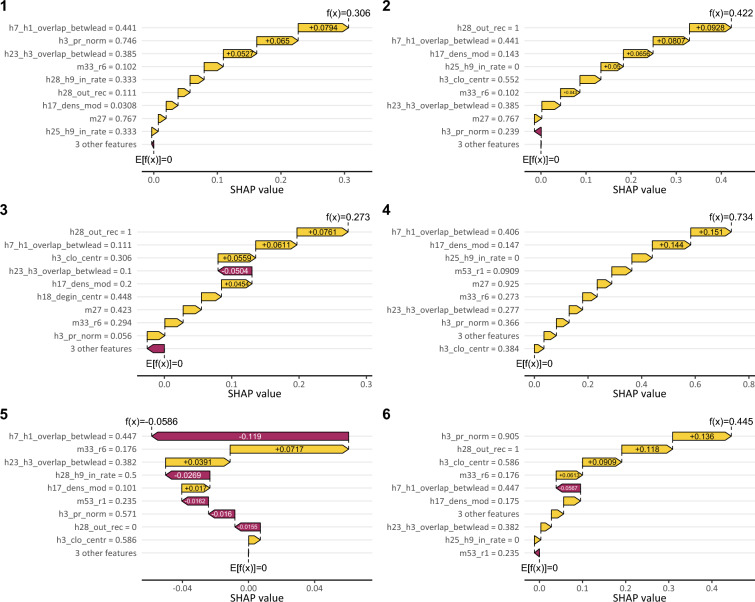
Degree of contribution of variables to the classification by cluster. The numbers at the y-axis indicate the maximum feature values of variables. The order of variables demonstrates the contribution of variables in case of each cluster.

**Figure 5:**
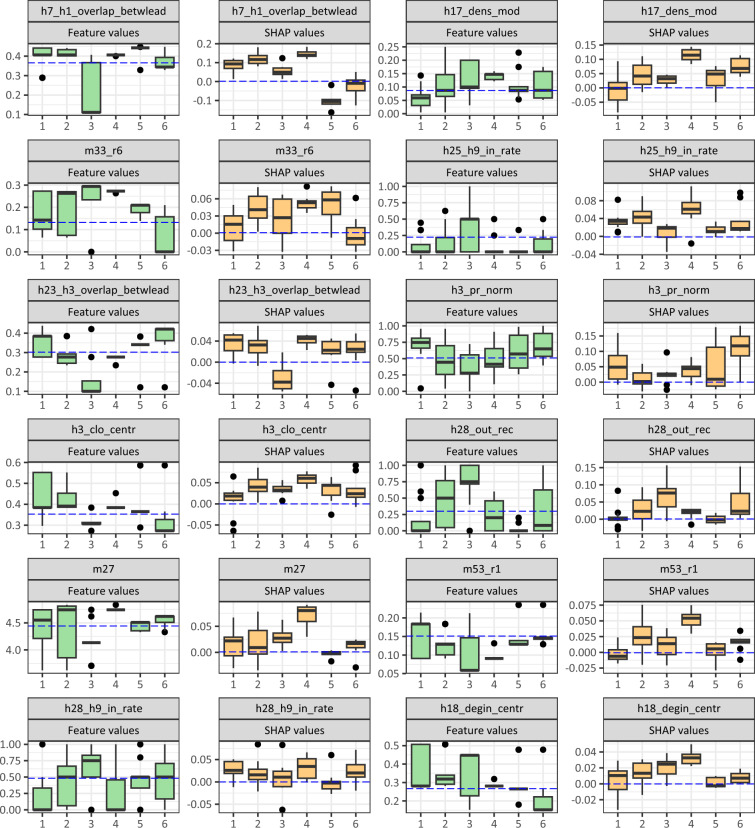
Distribution of variable values and SHAP values by cluster. The dashed blue line shows the mean values of the stayers in the figure.

Based on individual SHAP values, we were able to identify clusters in which the variables contributed to the prediction of the members in a similar way. Since in the present work we were interested in the background factors of quits, we searched for background factors among the 70 main quitters. We divided the quitters into 6 consistent groups based on the Silhouette method detailed in the Methods section. In this section, we characterize the clusters and present typical termination factors. Figure 4 shows the contribution of variables to the prediction in clusters. It can be seen that the contribution of the variables is not the same in all groups, and the distribution of the importance of the variables in Figure 5 varies between clusters.

The SHAP values and the feature values of the variables can provide good information to evaluate the clusters. SHAP values show how much a variable contributes to the prediction, but the feature value takes individuals in the variable and distinguishes the data points from each other. The distribution of SHAP feature values of the variables in each cluster is plotted together in the figure. The order of variables represents the importance of variables for all quitters. The number of persons belonging to a cluster is informative because the sample does not represent SMEs.

The resulting clusters are listed below and explained in detail in the Supplementary material.

*Cluster 1*: Peripheral actors in organizations with poor performance on several variables (13 persons)

*Cluster 2*: Hierarchy avoiders (18 persons)

*Cluster 3*: Victims of leadership dysfunctions (9 persons)

*Cluster 4*: Actors considered unfriendly (10 persons)

*Cluster 5*: Others (9 persons)

*Cluster 6*: Peripheral actors in organizations with good performance (11 persons)

The key findings are consolidated in Table 3, revealing distinct variable importance across SHAP clusters. Notably, the ranking of variables in the RF model differs significantly from the SHAP importance ranking derived from the logit model. Additionally, the table provides information on the representation of each organization surveyed within the importance groups. The cross-table of SHAP importance clusters and firms demonstrates a high significance level, evidenced by a $\chi ^2$ value of 187.68 and a *p*-value less than 2.2e-16. This underscores the strong firm-dependent nature of turnover determinants.

**Table 3:** Summary of the main results.

Variable	The level of variable	Rank of variables overall and in clusters by SHAP importance									
		Overall	Cluster 1	Cluster 2	Cluster 3	Cluster 4	Cluster 5	Cluster 6	Importance in logit	coefficients in logit	*p*-value in logit
h7_h1_overlap_betwlead	Organizational	1	1	2	2	1	1	5	8	$-$8.655	$0.1>$
h17_dens_mod	Mezo structural	2	6	3	5	2	5	6	12	$-$0.110	$\sim 1$
m33_r6	Organizational	3	4	6	8		2	4	6	7.718	0
h25_h9_in_rate	Individual	4		4		3			7	$-$2.113	0
h23_h3_overlap_betwlead	Organizational	5	3	7	4		3		3	10.994	0
h3_pr_norm	Individual	6	2		9		7	1	10	0.766	$0.1>$
h3_clo_centr	Organizational	7		5	3			3	1	22.793	$0.001>$
m27	Organizational	8			7				5	$-$2.361	0
h28_out_rec	Individual	9		1	1		8	2	9	0.960	$0.001>$
m53_r1	Organizational	10				4	6		4	$-$27.803	0
h28_h9_in_rate	Individual	11	5				4		11	$-$0.216	$\sim 1$
h18_degin_centr	Organizational	12			6				2	$-$13.415	$0.001>$

		Organizations represented by quitters in SHAP importance clusters (individuals in clusters)									
		Organization id	Cluster 1	Cluster 2	Cluster 3	Cluster 4	Cluster 5	Cluster 6			
		1			2						
		2		5		1					
		3			1			7			
		4									
		5	3								
		6			5						
		7	1	5							
		8					1	1			
		9					2	1			
		10					5	3			
		11	5	3							
		12	4	5	1	9					

The upper part of the table shows the ranking of the overall and cluster specific importance of the variables in the fitted RF model. The coefficients and significance of the variables in the logit model are included for comparison. The lower part of the table is a cross-table showing the distribution of surveyed companies within the SHAP variable importance clusters.

## Ethical statement

Participation in the research involved completing a questionnaire. Respondents voluntarily participated in the research and they were informed consent prior to their participation. The participants were informed that, due to the methodological nature of the network research, the data is collected by name, however, personal data will not be used when analyzing the data. After data collection, the names were hashed for data analysis. Network indicators and individual indicators anonymously provide sufficient information for research results and conclusions. The organizational operation questionnaire was filled out anonymously by the participants, and personal data was not recorded. We did not collect demographic information, only perceptions and feelings were measured, which were aggregated by organizations. The network data collection and the organizational operational data collection cannot be connected at the level of the respondents respecting the personal rights of the respondents. The research was carried out following the procedures outlined by the Declaration of Helsinki. All researchers worked according to the protocols declared in Code of Ethics of the University of Pannonia, Veszprém, Hungary. The research design followed the guidelines of the Code of Ethics. Ethics approval by the institutional committee (Ethics Committee of University of Pannonia) is not required specifically for this research. Personal data is adequately protected, making it impossible to infer personal consequences. Trends and patterns were the focus of the research, which only make it possible to characterize a certain population. For this type of research, the Code of Ethics states that the Ethics Committee does not require prior approval of the research. The authors declare that there are no ethical issues with the results presented.

## Discussion

Following the data pipeline steps represented in Figure 1, we retrospectively reconstructed the possible reasons for employee turnover in the organizations studied. The study identified 12 variables that best predict turnover. These variables include individual and organizational indicators related to skills, competencies, leadership, delegated workload distribution, decision-making, role in the communication network, and professional and personal relationships. It is worth noting that the organizational-level indicators can be interpreted as categorical variables since they can only take 12 different values. However, they still provide a good characterization of the working environment of the network actor through the ratings of the whole group.

In general, the characteristics of the networks of the firms and their members in the survey suggest that those loosely connected to the cooperation system and those on the periphery are at risk of leaving, but it is not true for all organization. As inconsistencies were also found between the results, suggesting a parallel phenomenon in the data, the quitters were further separated into groups to provide deeper insights. The clusters are based on SHAP values, and six consistent clusters are identified with similarly contributed variables in their classification.

Clusters showed that we can interpret several types of periphery that led to leaving the organization. On the one hand, those at the margins, are clearly measured by the centrality indicator. The periphery also includes those who are considered unfriendly, those who do not have friendly contacts, those at the margins of the communication network, those who lack professional information flow, and those who do not receive sufficient professional support from their managers. It is also peripheral if someone is perceived as less of a key person by his or her colleagues.

A group emerged who had high professional knowledge and were considered key people by colleagues, as well as ranked in a central position, but did not work well with their manager. They are likely to be successfully targeted by recruiters with whom they have developed an external network.

Moreover, the factors influencing turnover exhibit variations among different organizations, implying the absence of a universal model applicable to all. Further exploration in the future requires a more multi-firm database to delve deeper into these distinctions.

## Conclusion

This work aimed to develop a data analysis pipeline that can provide detailed information to decision-makers by uncovering the background factors for classification. The methodology is suitable to achieve aim, because

- Can perform with unbalanced classes,
- Can handle complex systems with many variables by filtering out the strongest predictors on a data basis,
- Use a state-of-the-art SHAP value based explanation approach,
- uncover parallel phenomenons in the background of a class with clustering of SHAP values.

The results obtained with this methodology contribute to the topic studied with new findings. The results suggest that the identified variables can be used to develop a reliable mathematical model to predict turnover. Whitening the black box model using SHAP values shows which variables contributed to the successful prediction. Clustering based on SHAP values showed specific pathways to positive class membership. The model has a limitation because it is based on the data available at the time of the study and may need to be updated as new data become available with more organizations.

Examining the substantial disparities in the SHAP importance of variables computed on data fitted with RF and logit models remains a prospective avenue for future research.

### Supplementary Information

Supplementary Information.

## Data Availability

The raw data analysed during the current study are not publicly available, as it was collected primarily for business interests, but are available from the corresponding author on reasonable request.
