# Supplementary material for: Explainable prediction of node labels in multilayer networks: a case study of turnover prediction in organizations
Source: Sci Rep. 2024 Apr 19;14:9036. doi: 10.1038/s41598-024-59690-4 (PMC11031594; doi:10.1038/s41598-024-59690-4)
Supplement: Supplementary file 1 — Supplementary Information. [file 41598_2024_59690_MOESM1_ESM.docx]

# Supplementary materials

## Descriptions of selected variables

Rigorously following the feature selection steps described in the methodology, we obtained by a data-driven approach the 12 variables for the model that best predict the classes:

- **m33_r6**: The organization level indicator reflects the extent of the leadership skills of managers. The rate of answer 6 (strongly agrees) to the question 'The management is adequately qualified to lead.'
- **m53_r1**: The organizational level indicator reflects with the level of dissatisfaction the extent to which employees perceive the workload distribution to be equal. The rate of answer 1 (strongly disagrees) with the question 'Everyone in the organization is equally burdened with work.'
- **m27**: It shows the appropriateness of management's assignment of tasks. The organizational average of the question "Managers assign tasks correctly."
- **h7_h1_overlap_betwlead**: This organizational level indicator reflects the depth of professional cooperation between managers. The likelihood of overlapping edges of the professional advice network between managers with feedback. It is likely that when a manager asks a peer manager for professional advice, he or she also receives feedback from the same manager. Feedback is a reinforcement that motivates and increases commitment.
- **h23_h3_overlap_betwlead**: An indicator for the organization that refers to massive conflict situations between managers. The likelihood that communication relationships between leaders are associated with the leader perceiving his/her leader partner as having good leadership skills. In other words, how likely is it that when communicating with someone, one is also likely to perceive the communicating partner as having good leadership skills. (Background research was carried out taking into account the interview research on which indicator of the network changes when there is some deep conflict between leaders, and they blame each other. We found that they were significantly less likely to rate their peers as good leaders. While sympathy-type relationships are maintained in the extent.)
- **h3_clo_centr**: The organization indicator refers to the degree of hierarchy in the communication network. Freeman centralization is calculated on the basis of the closeness centrality of communication network actors. The value is high if a few actors are centrally located while the rest of the network is not in a communicative relationship. If the communication paths are close to random, the value is low.
- **h18_degin_centr**: An organizational indicator that gauges the decentralization or centralization of decision-making. In network research, respondents nominate staff or managers whom they perceive to be making crucial decisions. In the resulting decision-making "network," we tallied the incoming degrees of all individuals and computed the Freeman centralization for each organizational network, yielding a specific categorical variable. A high value implies that decisions are concentrated within a narrow group, while a low value indicates a more distributed decision-making process throughout the organization.
- **h17_dens_mod**: Density of coordination activity within the local community. Selecting those in the network who facilitate proper operation is possible. Within the Leiden modules identified in the collaboration network, we calculated the density of edges directed at those who contribute to smooth functioning in the modules.
- **h3_pr_norm**: The importance of actors in the communication network is expressed with centrality. Min-Max normalized PageRank centralities in the communication network are in the variables to ensure network comparability. 0 indicates a central position close to the origo, and 1 indicates a peripheral position.
- **h28_out_rec**: Percentage of reciprocal outgoing friendship connection. When asked whom one would like to spend lunch with, any staff or managers can be selected. Mutual relationships imply a deeper relationship. The individual-level variable expresses the rate of reciprocal or mutual connections and all outgoing friendship relationships.
- **h25_h9_in_rate**: The individual-level indicator shows the proportion of collaborators who consider him or her a key person. The likelihood that incoming collaboration relationships cooccurred with a key person consideration, i.e., the overlap of the collaborative and key person network in someone's incoming nodes.
- **h28_h9_in_rate**: Very similar to the previous one. It shows the proportion of collaborators who consider him or her a friend. The likelihood that incoming collaboration relationships cooccurred with a friendship consideration.

## Details of SHAP clusters

**Cluster 1**: Peripheral actors in organizations with poor performance on several variables (13 persons)

The periphery can be interpreted in several ways. The cluster has low centrality in the communication network based on the normalized PageRank value (**h3_pr_norm**). Their importance position in the organization is, on average, the lowest 25\%. For those in the cluster, this indicator is the worst compared to all other leavers, much worse than those remaining in the organization. In addition, it is characteristic that the communication network in an organization is hierarchical (**h3_clo_centr**) (similar to Cluster 2). It is centralized in those with decision-making ability (**h18_degin_centr**), i.e., a narrow group can make decisions in the organization. Many people in their organization (**m53_r1**) have a sense of justice and do not feel equally burdened by their colleagues. Those in the cluster are less likely to be befriended by their peers, have low co-marking of friendship for cooperative partners, and report less co-marking of friendship.

**Cluster 2**: Hierarchy avoiders (18 persons)

The group includes employees rated well on individual and organizational indicators. However, one organizational indicator, centralization of the communication network (**h3_clo_centr**), which is considered, is an important predictor. In their case, it is the highest compared to other quitters and stayers, which means it can certainly play a role in their exit. The centrality of the communication network slows down communication paths; information gets to the right place more slowly than in a flat organization. Another important distinguishing factor of this cluster is the key person status. People in cluster 2 are not perceived as key people by their collaborating partners (**h25_h9_in_rate**) according to their incoming cooperative connections that overlap with the perception of key people. That is, they are not considered key people by the cooperating partners. Only one person out of 18 is recognized as a key person by their collaborators with a probability of 62.5%. The status of the key person can also be seen as a peripheral role but is likely to be associated with professional, workflow components, or factors of influence similar to humans.

**Cluster 3**: Victims of leadership dysfunctions (9 persons)

Cluster members have excellent individual indicators. They are considered very friendly by their colleagues, both in terms of the overlap of friendship with their incoming cooperation relationship (**h28_h9_in_rate**) and the reciprocity of their outgoing friendship connections (**h28_out_rec**). They play an important role in the communication network according to Pagerank centrality (**h3_pr_norm**). Their collaborative partners also consider them key people (**h25_h9_in_rate**). That is, they can be seen as embedded in the functioning of the organization. However, the leadership variables in the set indicate a poorly led organization. In their case, the professional collaboration of managers is the least complex (**h7_h1_overlap_betwlead**). Leaders who seek professional advice from other leaders are less likely to receive feedback from their peers. The delegation of tasks by leaders is not well respected, as indicated by the low characteristic value of the variable **m27**. Managerial decision-making is centralized, with a narrow range of managers capable of making decisions indicated by the high value of the characteristic **h18_degin_centr**. However, they work in organizations where managers are less likely to rate their leader peers as having good leadership skills (**h23_h3_overlap_betwlead**), which is an information trace of the hidden conflicts between leadership, which is already noted above. However, this phenomenon is associated with a negative SHAP value, suggesting that this is not why they leave their SME. In addition to all this, the leadership skills of managers are rated excellent (**m33_r6**) by a high proportion of their organizations. It is indicative of the complexity of leadership that while overall, they consider their leaders to be good leaders on average, inadequate task delegation as a leadership function still leads to an exit. This exit path is most true for those doing white-collar work. Empowering leadership is common in IT and engineering firms, where problem-solving is delegated to employees. Not everyone seems able to cope with the empowered leadership style and feels the need for more management involvement in problem-solving. The roles should be clear in the cooperation between the employee and the leader in achieving a successful organizational outcome.

**Cluster 4**: Actors considered unfriendly (10 persons)

They work in organizations that have good indicators of leadership. The quality of task delegation (**m27**), leadership competence of leaders (**m33_r6**), professional cooperation between leaders (**h7_h1_overlap_betwlead**) are excellent. The distribution of tasks among employees is rated fair (**m53_r1**). However, there are individual indicators to which exit can be related. The reciprocity of outgoing friendship connections (**h28_out_rec**) and the overlap of friendship and collaboration connections (**h28_h9_in_rate**) are low in this cluster. They are neither peripheral nor central in terms of PageRank centrality in the communication network.

**Cluster 5**: Others (9 persons)

The most uncharacteristic cluster. Most indicators are similar to those of the stayers or indicate a well-functioning organization. Two individual indicators show some correlation with quitting the organization. They are less likely to be considered key people by those who work with them (**h25_h9_in_rate**). They are also less likely to have a mutual friendship relationship (**h28_out_rec**). Therefore, they are similar to those in Cluster 4. However, in Cluster 4, an important predictor is the lack of friendly layer overlap with collaboration (**h28_h9_in_rate**), which is not the case for those in this cluster. It is assumed that they would rather be friends with others than those with whom they cooperate, but this intention is not reciprocated.

Cluster 6: Peripheral actors in organizations with good performance (11 persons)

One of the most important predictors is the less important role in the communication network based on PageRank centrality (**h3_pr_norm**). In this sense, it is very similar to those in Cluster 1. However, they are quit from organizations whose indicators are sufficient. The communication network structure is not hierarchical (**h3_clo_centr**). Managers value each other's leadership skills, and there is no indication of conflict (**h23_h3_overlap_betwlead**). Leadership competence of leaders was rated significantly lower proportion as excellent (**m33_r6**), but in their case this variable has a negative SHAP value, which can be assessed as not related to leaving the organization in this cluster.
